# Animal models of fungus-related allergic airway disease: exposures, readouts, mechanisms, and translational gaps

**DOI:** 10.3389/fcimb.2026.1856761

**Published:** 2026-05-28

**Authors:** Yinghui Qu, Ruichen Du, Peize Liu, Yun Pan, Lili Zhi, Yonghua Gao, Qian Qi

**Affiliations:** 1 Department of Respiratory, The First Affiliated Hospital of Shandong First Medical University & Shandong Provincial Qianfoshan Hospital, Shandong Institute of Respiratory Diseases, Jinan, Shandong, China; 2 Shandong Provincial Key Medical and Health Laboratory of Translational Medicine in Microvascular Aging, The First Affiliated Hospital of Shandong First Medical University & Shandong Provincial Qianfoshan Hospital, Jinan, Shandong, China; 3 Department of Allergy, Shandong Provincial Qianfoshan Hospital, Shandong Institute of Respiratory Diseases, The First Affiliated Hospital of Shandong First Medical University, Jinan, China; 4 Department of Respiratory and Critical Care Medicine, Shanghai Pulmonary Hospital, Tongji University School of Medicine, Shanghai, China

**Keywords:** allergic airway disease, allergy, animal model, asthma, fungal, fungal sensitization

## Abstract

Fungal sensitization is a major risk factor for severe asthma and related airway diseases, spanning presentations from simple sensitization to allergic bronchopulmonary aspergillosis (ABPA). To capture this spectrum, the concept of allergic fungal airway disease (AFAD) has been proposed. Animal models have been central to uncovering how fungi drive airway inflammation. Extract-based models are simple and reproducible and highlight epithelial alarm signaling and type 2 immunity, although they do not reflect chronic infection. Spore-based models using *Aspergillus fumigatus* or *Alternaria alternata* more closely mimic human disease, producing mixed eosinophilic and neutrophilic inflammation, airway hyperreactivity, and mucus plugging. Advances using clinical isolates, gene-edited fungal strains, and combined exposure systems (for example, cigarette smoke plus spores) have further improved relevance by linking fungal virulence and host factors to disease severity. This review summarizes available models, their strengths and limitations, and their utility in elucidating AFAD pathogenesis and informing therapy.

## 1 Introduction

Fungal sensitization is an increasingly recognized contributor to chronic airway disease. Sensitization to *Aspergillus fumigatus*, *Alternaria alternata*, and other environmental fungi is common in patients with severe asthma, and it is associated with poor symptom control, frequent exacerbations, and accelerated loss of lung function (Kao et al., 2021; Tiwary and Samarasinghe, 2021; Treadwell et al., 2024). At the most severe end of this spectrum, allergic bronchopulmonary aspergillosis (ABPA) is characterized by markedly elevated IgE, recurrent pulmonary infiltrates, and bronchiectasis (Roboubi et al., 2023). To capture this heterogeneity, the concept of allergic fungal airway disease (AFAD) has been proposed, describing a continuum that ranges from simple sensitization to destructive airway pathology (Pashley and Wardlaw, 2021; Wardlaw et al., 2021). Despite the strong epidemiological and clinical associations, the mechanistic pathways linking fungal exposure to persistent inflammation and airway remodeling remain incompletely understood.

Animal models have been central to progress in this field. Early experimental approaches used intranasal administration of fungal extracts or culture filtrates. These systems are technically straightforward and reproducible, and they reliably induce type 2-dominated inflammation, including eosinophilia, elevated IgE, and mucus hypersecretion. They have been crucial for identifying epithelial-derived cytokines such as IL-33 and thymic stromal lymphopoietin (TSLP) as upstream mediators of type 2 immunity. However, extract-based models lack viable fungi and cannot simulate chronic persistence or the immunological effects of fungal cell wall structures.

To address these limitations, models using live conidial exposure were developed. Repeated intranasal or aerosol administration of *A. fumigatus* or *A. alternata* spores elicits mixed granulocytic inflammation, airway hyperresponsiveness (AHR), and mucus plugging, more closely resembling clinical disease. Certain clinical isolates, such as *A. fumigatus* strain W72310, can persist in murine lungs for several weeks, inducing recurrent inflammation that parallels relapsing ABPA. Comparative studies with Alternaria have also shown that spores and filtrates are not equivalent stimuli: filtrates primarily drive Th2 responses, whereas spores additionally activate IL-17 pathways, which may explain the steroid resistance observed in some patients. Recent advances have further refined these systems. Gene-edited fungal strains, such as Alternaria mutants lacking pore-forming proteins, have demonstrated that epithelial membrane damage is required for allergic sensitization. Other work has incorporated host risk factors; for example, combining cigarette smoke exposure with *A. fumigatus* spores models fungal exacerbations in chronic obstructive pulmonary disease (COPD). Together, these approaches illustrate how fungal pathogenicity interacts with host background to shape disease severity. Current fungal allergy models therefore span a wide methodological range, from extract-based systems that emphasize epithelial alarm signaling to spore-based models that recapitulate chronicity, genetically defined strains that enable causal testing of virulence factors, and combined exposure models that reflect clinical comorbidities. Each offers specific strengths and limitations, and collectively they provide complementary insights into AFAD mechanisms. This review synthesizes available animal models, compares their design and readouts, and assesses their relevance for understanding disease pathogenesis and informing therapeutic development. To support systematic comparison, the main models cited in this review and their key characteristics are summarized in the following section (**Table 1**).

**Table 1:** Summary of fungal sensitization animal models.

Disease type	Fungal species & strain	Exposure route & dose	Animal type & age	Immune phenotype	Key readouts	Mechanistic insights	Clinical relevance	Simulated clinical stage	Key references
Fungal extract–based models	*Aspergillus fumigatus extract (item XPM3D3A25, lot* *365084; Stallergenes Greer)*	Mice were treated with 50 mg of ASP once a week for 4 weeks followed by 20 mg of ASP twice a week for 4 weeks	hβcTg mice 8–12 weeks old	Mixed eosinophilic–neutrophilic, Th2 + Th17	AHR, Collagen deposition (Masson trichrome staining), Abnormal lung function, airway remodeling	This type of mouse responds to GM-CSF and IL-5, but not to IL-3	hβcTg mice ensure specific binding of CSL311 to human β c receptors	Severe Airway Remodeling	(Wang et al., 2024)
Fungal extract–based models	*Aspergillus fumigatus crude protein extract (Greer Laboratories, Lenoir, NC, USA)*	Inject 200 ug AF cpe intraperitoneally on days 0 and 14, and nebulize and inhale aerosols containing AF cpe on days 28, 29, and 30	CFTR S489X -/-; FABP-hCFTR(Whitsett mouse) 5–6 weeks old	Mixed eosinophilic–neutrophilic, Th2 + Th17	High IgE, PAS^+^ staining	Using CFTR ^489^ X ^-^/^-^ and FABP hCFTR ^+^/^+^ hybrid mice	Key characteristics of ABPA in simulated CF patients	Sensitization	(Mueller et al., 2009)
Fungal extract–based models	*Aspergillus fumigatus (from Bayer Pharmaceuticals)*	50 μL of Aspergillus antigen was applied to the left nare. Mice were immunized three times a week for 3 weeks	129/SVEV	Eosinophil-mediated type 2 inflammation	High IgE, AHR, eosinophil infiltration	Repeated intranasal administration of *Aspergillus fumigatus* extract and use mice with a null mutation of the C locus	IgE is not required for allergen-induced BHR	Sensitization	(Mehlhop et al., 1997)
Fungal extract–based models	*Aspergillus fumigatus*	Intranasal administration of 50 μ-g AA filtrate + PBS on days 0, 3, and 6, sensitization with 20 μ-g AF extract+ adjuvant on days 0 and 7, and intranasal administration of 25 μ-g+ PBS on days 12, 13, and 14	BALB/cand C57BL/6 8–10 weeks old	Eosinophil-mediated type 2 inflammation	eosinophil infiltration, HE staining, Immunofluorescence staining	Eosinophils survive ex vivo in the absence of exogenous pro-survival cytokine support, the long-term persistence of eosinophils in the airway depends on IL-13	Eosinophils are phenotypically and functionally heterogeneous.	Sensitization	(Geslewitz et al., 2018)
Fungal extract–based models	*Aspergillus fumigatus(A1160 and its derived △5 and △13 mutant strains)*	25µl of culture filtrate was administered by intranasal instillation twice a week for five weeks	BALB/c 8 weeks	Eosinophil-mediated type 2 inflammation	High IgE, AHR, Th2 cytokines, cell counting, PAS + staining	Using genetically modified strains	Clarify the specific role of a single protease in the induction of airway diseases by *Aspergillus fumigatus*	Severe Airway Remodeling	(Namvar et al., 2015)
Fungal extract–based models	*Alternaria alternata*	A.alternata was administered intranasally 3 times per week (5 mg for the first 2 weeks, followed by 10 mg in the third week).	BALB/c or ST2-/- 3 days	Eosinophil-mediated type 2 inflammation	High IgE, high IL-33, AHR	Steroid-resistant AHR, Simulate childhood asthma	IL-33 might be a novel therapeutic target for SAFS.	Sensitization	(Castanhinha et al., 2015)
Fungal extract–based models	*Aspergillus fumigatus extract(from Greer Laboratories)*	Intraperitoneal injection of Af extract for stimulation. After one week, mice were given Af extract intranasally three times a week for four weeks	BALB/c or C3aR-/- 8 weeks old	Eosinophil-mediated type 2 inflammation	High C3a, C3d, deposition,	Local sinonasal C3 concentrations are associated with disease severity	therapeutic potential of local sinonasal complement inhibition as a means to modulate local inflammation and the development of CRS	Severe Airway Remodeling	(Mulligan et al., 2018)
Fungal extract–based models	Aspergillus niger NIgA2 and NIgB1	Recombinant perforin proteins NIgA2 and NIgB1 (24 μg each) were administered intranasally on days 1, 4, 7, 10, and 13, and analysis was performed on day 15	C57BL/6 wild-type mice, 5–8 weeks old	type 2 inflammation	Flow cytometry: Quantification of lung Th2 cells and eosinophils ELISA: Total serum IgE concentration	Cross-species conservation: A. niger homologous perforin proteins also induce type 2 immunity Testing “membrane perforation is a universal initiation signal”	Explain the common mechanisms underlying allergies to different molds and broaden the applicability of therapeutic targets.	Sensitization	(Shi et al., 2025)
Live fungal spore models	*A. fumigatus*	After 7 days of antigen sensitization + 3 intranasal attacks, intranasal intratracheal inoculation of 5.0 × 10^6^ live spores was administered	Wild-type BALB/c (Stat6+/+) mice, Wild-type BALB/c (Stat6-/-) mice	Th2-dominated (elevated levels of IL-4 and IL-13) chronic inflammation (lymphocyte-dominated, accompanied by eosinophils, etc.) has both Stat6-dependent and Stat6-independent characteristics.	AHR, peribronchial fibrosis (collagen deposition), goblet cell hyperplasia, and mucus hypersecretion	Chronic Stat6 signaling bypass drives persistent pathology.	Stat6 *in vivo* research platform for non-dependent fungal sensitized asthma.	Sustained Colonization	(Blease et al., 2002)
Live fungal spore models	*Alternaria alternata* (strain 18586), *Cladosporium herbarum* (strain 19275)	intranasal inoculation; The dose is 2×10^5^ spores or 5 μg extract (dissolved in 100 μL PBS) twice weekly (Monday and Friday) for 10–12 weeks.	Female BALB/c mice	Th2 immune response: elevated serum total IgE, specific IgE and IgG1 production, elevated lung IL-4, IL-5, IL-13, TGF-β	Increased inflammatory cells (macrophages, neutrophils, lymphocytes, eosinophils), AHR, increased markers of pulmonary fibrosis (hydroxyproline, collagen, fibronectin), histology showing epithelial thickening, goblet cell hyperplasia, increased mucus secretion, subepithelial fibrosis.	Chronic intranasal exposure to mold allergens does not require adjuvant sensitization, that is, its protease activity and β-glucan directly break airway tolerance, driving Th2 inflammation and IL-13/TGF-β-mediated airway remodeling.	Highly simulates human mold-induced chronic allergic asthma and airway remodeling.	Severe Airway Remodeling	(Denis et al., 2007)
Live fungal spore models	Live Aspergillus niger spores	Intranasal inoculation, live spores (4 × 10^5^) + LPS (1 μg), 3 times a week for a total of 8 times.	Female, C57BL/6J,5–8 weeks	Th1/Th17 is dominant, and Th2 is inhibited; Neutrophil infiltration, CD8^+^ T cell increase; Lung parenchyma noncompact granulomas.	Decreased hyperresponsiveness of the airways; eosinophilia; IgE persistent; Granulomas form in the lung tissue.	High dose LPS inhibits fungal-induced Th2 responses and enhances Th1/Th17 responses; Live fungi synergistically induce HP-like pathological changes with LPS.	HP models were provided through fungal infection + high-dose LPS exposure	Sensitization	(Zeng et al., 2021)
Live fungal spore models	*Alternaria alternata*, ATCC 66981	Intranasal inoculation: live spores (10^5^) or filtrate (10 μg) once a week for 6 weeks	BALB/c mice, Adult, 5 weeks; Neonatal rats, 1 week old	Filtrate: strong Th2 reaction (EOS↑, IgE↑↑), with Th1 type antibody (IgG1↑), no Th17 reaction. Spores: Th2/Th17 coexist (eosinophils↑, IL-17↑), with neutrophil inflammation, IgE↑, IgG1↑ (weaker than filtrate)	Enhanced AHR; inflammation of lung tissue; Mucous cell metaplasia (MCM)	Spore induction is closer to true exposure, triggering neutrophil-to-Th17 responses; The filtrate induces a stronger Th2 reaction with antibodies	It provides an animal model that is closer to real exposure for studying Alternaria-induced asthma (asthma in adults and children).	Sensitization	(Daines et al., 2021)
Live fungal spore models	*Aspergillus fumigatus*, ATCC 13073	Endotracheal inoculation. Acute: 7×10^7^ conidia per dose; Chronic: Day 0, 1 × 10^7^ live spores. On days 7-11, 14-16, 1 × 10^6^ live spores per day.	C57BL/6NTac mice, WT and Chia^-^/^-^ (AMCase-deficient) mice	Acute: Th1/Th2/Th17 mixed↑; Chronic: Th17 specific↓, Th2 unchanged, lung function improved	Acute: Reduced pulmonary fungal load (48 hours) Chronic: improved AHR, decreased airway resistance, decreased goblet cell hyperplasia/mucus secretion, and unchanged pulmonary fungal burden	The bidirectional role of AMCase in acute and chronic fungal infections was revealed: negative regulator (inhibition of host defense) in acute infection and pathogenic factor (aggravation of disease) in chronic asthma.	A mouse simulation model was provided for chronic ABPA, and AMCase was suggested as a potential target	Severe Airway Remodeling	(Garth et al., 2018)
Live fungal spore models	*Aspergillus fumigatus* (CEA10)	Intranasal inoculation, 4 × 10^5^ live spores per dose, repeated 3, 6, or 9 times over 19 days.	Immunocompetent C57BL/6 mice; various transgenic/knockout mice (e.g., Il13eGFP, Il17Cre ROSAeYFP, Cd11c. DOG, Mgl2-DTR, etc.).	Type 2 inflammation specifically driven by Mgl2+ cDC2s	Inflammatory cell counts; cytokine levels (IL-13, IL-17); single-cell sequencing/flow cytometry tracing of DC subsets; pathology (epithelial thickness, goblet cells).	Mgl2+ cDC2s specifically drive Type 2 inflammation (depletion results in a reduced Type 2 response, while Type 17 remains unchanged); CD4+ T cells are essential for both Type 2 and Type 17 responses.	Reveal Mgl2+ cDC2s as a potential novel therapeutic target for the Type 2 inflammatory endotype of fungal asthma.	Sensitization	(Cook et al., 2025)
Live fungal spore models	*Aspergillus fumigatus*, ATCC 13073	On day 0, 1 × 10^7^ live spores. On days 7-11, 14-16, 1 × 10^6^ live spores per day.	C57BL/6NTac mice, WT, Dectin-1 deficient , IL-22 deficient; 6–8 weeks old	Th2/Th17 mixed inhibitory type, IL-22 drives inflammation	Dectin-1/IL-22 deletion: improved airway hyperresponsiveness (AHR), decreased pulmonary resistance, goblet cell hyperplasia/decreased mucus, decreased inflammation in lung tissue, and unchanged lung fungal burden.	The pathogenic role of the Dectin-1/IL-22 axis in fungal asthma was revealed	The repeated live spore exposure model does not rely on adjuvant sensitization and is closer to the natural human sensitization process.	Sustained Colonization	(Lilly et al., 2012)
Live fungal spore models	*Aspergillus fumigatus*, Clinical isolates 293	Endotracheal inoculation: 10^8^ spores (live/heat-inactivated spores) in a single dose	C57BL/6J female mice, 6–8 weeks old	Live spore group: Th1 (IFN-γ), with isotype conversion antibodies (IgG1, IgG2a, IgG2b) production Heat-inactivated spore group: Th2 (IL-4/IL-13), no isotype converting antibody production	Recruitment and proliferation of CD4^+^ T cells; antibody response; GMS staining showed sterile filament growth in the lungs	Spore viability determines immune bias and antibody response	The live spore model is suitable for evaluating protective vaccines; The dead spore model is suitable for studying allergen immunotherapy	Sensitization	(Rivera et al., 2005)
Live fungal spore models	*Aspergillus fumigatus* ATCC Strain 13073	Endotracheal inoculation. On day 0, 1 × 10^7^ live spores. On days 7-11, 14-16, 1 × 10^6^ live spores per day. Purified chitin exposure model: endotracheal inoculation, 50 μg/time, excitation mode is the same as that of live spores	C57BL/6 WT mice, genetically modified mice: AMCase deletion type (Chia^-^/^-^), AMCase overexpression type (SPAM), 6–12 weeks old, male and female	Live spore model: Typical Th2/Th17 inflammation with airway hyperresponsiveness (AHR) Purified chitin model: induces moderate mixed inflammation	Lung resistance increased and inflammatory cell subsets increased significantly. TH2/TH1/TH17 related factors were significantly increased, and chitin-positive spores were detected in the lungs	The use of highly purified chitin proved that it cannot trigger AHR alone, chitinase activity is a key regulator, and the real effect may be to degrade chitin in the spore cell wall, thereby exposing or releasing other pathogenic fungal components (such as β-glucan), which are necessary to drive AHR.	A highly controllable chronic exposure model for isolating a single fungal component (chitin) was established.	Sensitization	(Ellis et al., 2024)
Live fungal spore models	*Aspergillus fumigatus* (strain INH5223).	dry aerosol inhalation; live/dead spores; 10 minutes once a week × 3 weeks	BALB/c mice	Non-allergenic group: Only live spores cause strong Th2 inflammation. Sensitization group: Both live and dead spores caused significant Th2 humoral responses, as well as neutrophil infiltration and goblet cell metaplasia	Humoral antibodies, inflammatory cell counts, histopathological staining, qPCR Goblet cell metaplasia, epithelial layer thickening, collagen deposition, local and systemic antibody reactions, TH2-related gene expression	Spore viability (germination) is key; The sensitization state amplifies the response to dead spores.	Non-invasive dry powder spore inhalation and simulated long-term repeated exposure are used to closely match the natural human sensitization process.	Sensitization	(Pandey et al., 2013)
Live fungal spore models	*Aspergillus fumigatus* Wild-type strain (10AF) and protease-deficient mutant (*ΔprtT/ΔxprG*)	Intranasal inoculation with 105 conidia in 40 μl PBS every 3 days for 14 days.	CFTR-deficient mice 6–8 weeks	Th2 type allergic inflammatory	Serum IgE, Cytokine levels in lung homogenates, Eosinophil marker gene expression, Fungal burden	Iron priming enhances Aspergillus protease activity, which drives a stronger Th2 immune response.	Airway iron may represent a modifiable risk factor for ABPA in cystic fibrosis patients, suggesting that localized iron chelation could be a potential therapeutic strategy.	Sustained Colonization	(Chatterjee et al., 2026)
Live fungal spore models	*Aspergillus fumigatus*	Intranasal, 2 × 10^5^ live conidia, daily for 14 days	C57BL/6J mice (wild-type and IL-1RAP^-^/^-^ knockout)	Type 2 allergic inflammation	BALF cell counts, Cytokine levels, Lung fungal burden, Histopathology, Flow cytometry, RNA-seq and proteomics	IL-33/IL-1RL1/IL-1RAP axis drives type 2 inflammation via ILC2 activation, leading to IL-5, IL-13, and eosinophilia. IL-1RAP deficiency abrogates this pathway without affecting fungal clearance.	IL-1RAP is a promising therapeutic target for eosinophilic airway diseases. Monoclonal antibodies targeting IL-1RAP offer a strategy to block multiple IL-1 family cytokines simultaneously.	Sensitization	(Williams et al., 2026)
Combined allergen–fungal exposure models	*Alternaria alternata* CBS 103.33	Intranasal instillation *A. alternata* crude extract: 20 µg per day Purified native Alt a 1:5 µg per day Recombinant Alt a 1: 5 µg per day	BALB/c Female 6–8 weeks old	Eosinophil-predominant, Th2	Eosinophil in BALF, Cytokine levels, Serum total IgE,	Alt a 1 induces the release of epithelial IL-25 and IL-33, the potential activation of ILC2s Established a model using intranasal exposure only	provide an animal model for targeted immunotherapy for fungal allergic asthma	Sensitization	(Vélez-Del-Burgo et al., 2021)
Combined allergen–fungal exposure models	*Aspergillus fumigatus*(No specific type)	Inhalation (nebulization), Aerosolized spore suspension (1 × 10^6^ CFU/ml), 30 min/day, 5 days/week for 4 weeks.	Wistar rats. 5–6 weeks old Male	Th2-type eosinophilic inflammation	AHR, Eosinophil count and percentage in BALF, PGD_2_ levels in BALF/serum, CRTH2 mRNA/protein expression in lung tissue	Upregulates PGD_2_ production in the airways and Increases expression of its receptor CRTH2 in lung tissue, the PGD_2_/CRTH2 axis drives eosinophil recruitment into the airways.	Identifies the PGD_2_/CRTH2 pathway as a critical mechanism for fungal-induced asthma attacks and provides a therapeutic target for clinical use of CRTH2 antagonists	Sensitization	(Liu et al., 2014)
Combined allergen–fungal exposure models	*Aspergillus fumigatus* strain MF-13	Intranasal inoculation, 5×106 conidia per mouse each time, performed on days 19, 21, and 23	Female BALB/c mice, 4 weeks old.	Mixed eosinophilic and neutrophilic, Increased Th2 cytokines and chemokine MIP-2.	BALF cell counts and cytokine levels, Lung histopathology (HE and PAS staining), Phagocytic activity of alveolar macrophages against Af conidia.	Dexamethasone -suppressing IL-5- reduces eosinophilic inflammation Itraconazole-suppressing MIP-2- reduces eosinophilic and neutrophilic inflammation Dexamethasone could restores phagocytic function of alveolar macrophages in allergic mice.	differential roles for corticosteroids and antifungals in fungal-exacerbated asthma corticosteroids may not worsen antifungal immunity in the context of allergic inflammation.	Sensitization	(Matsuse et al., 2013)
Combined allergen–fungal exposure models	Extract of *Alternaria alternata*(from Greer Laboratories).	Intranasal instillation, single challenge with 10 µg (in 50 μL PBS)	Female BALB/c mice (wild-type) and ST2-deficient mice on a BALB/c background, 6–8 weeks old.	Th2-predominant inflammation with prominent innate immune activation.	Eosinophil/Neutrophil counts in BALF, Cytokines/Mediators in BALF, MCPT-1 in serum. Lung mucus score (PAS staining), MUC5AC gene/protein expression, Lung Function	axis: Alternaria protease → Activates PAR-2 → Promotes ATP release → Triggers rapid release of full-length IL-33 from lung epithelial cells → Th2 inflammation	provides a new theoretical target for the diagnosis and treatment of SAFS	Sensitization	(Snelgrove et al., 2014)
Combined allergen–fungal exposure models	Aspergillus extract	Intranasal instillation, 5 μg per challenge, Twice weekly for 8 weeks.	Female BALB/c mice. 12–15 weeks old	classic Th2 type allergic inflammatory phenotype with eosinophil/mast cell infiltration	AHR, eosinophilia/mastocytosis, mucus hypersecretion, smooth muscle hyperplasia, collagen deposition, Lung Th2 cytokine levels, serum-specific IgE/IgG1.	allergens synergistically activate the p38 MAPK signaling pathway, activating dendritic cell, providing co-stimulatory signals, driving sustained T cell activation	Mimics the clinical situation that most asthmatic patients are sensitized to multiple allergens.	Sensitization	(Goplen et al., 2009)
Combined allergen–fungal exposure models	*Aspergillus fumigatus* (from a patient with invasive aspergillosis)	Intranasal instillation; 1×10^4^ spores in 100 µL per administration, twice weekly for 1, 3, or 5 weeks	Male, Wistar rats, 220–250 g (young adult, not mentioned age)	Th2 type inflammation with elevated IL-13	MUC5AC protein and IL-13 levels in BALF, MUC5AC mRNA and protein expression in lung tissue, Goblet cell hyperplasia, AHR	Chronic *A. fumigatus* exposure → IL-13↑ → MUC5AC expression↑→ goblet cell hyperplasia ↑ → mucus hypersecretion →AHR↑ → exacerbation	fungal exposure may aggravate the disease through IL-13/MUC5AC pathway in no-ABPA asthma patients, suggest that IL-13 and MUC5AC are potential therapeutic targets	Severe Airway Remodeling	(Gao et al., 2012)
Combined allergen–fungal exposure models	*Aspergillus fumigatus* MF-13	Intranasal; 5×10^6^ live or dead conidia each time, given three times on days 19, 21, and 23.	Female BALB/c mice; 4 weeks old	Mixed eosinophilic and neutrophilic, Increased Th2 response	eosinophils and neutrophils in BALF; IL-5, IL-13, MIP-2 in lung tissue; Muc5ac expression; PAS staining; Gomori staining	Dead spores enhance Th2 response and mucus secretion; live spores additionally induce neutrophil inflammation by MIP-2	To simulate the clinical situation of asthma exacerbation caused by *Aspergillus fumigatus* as both an allergen and a pathogen	Sensitization	(Fukushima et al., 2010)

## 2 Fungal extract–based models

### 2.1 Model overview and sensitization principle

Animal models sensitized with filtrate extracts were first reported in the 1990s. Today, the filtrate extract–induced animal model is one of the most widely used approaches (Kurup and Grunig, 2002; Grunig and Kurup, 2003), as it can closely reproduce the core immunological features of human ABPA, producing a strong Th2 immune response. Fungal crude extracts are mixtures of culture supernatants and mycelial components with complex compositions. They contain more than 20 known allergens, such as Asp f 1, Asp f 5, and Asp f 13 (Kurup and Grunig, 2002; Grunig and Kurup, 2003; Namvar et al., 2015), and also various proteases, toxins (including gliotoxin and hemolysin), and other bioactive molecules. Among these components, proteases can directly damage the airway epithelium and disrupt the mucosal barrier. They are also considered natural adjuvants for Th2 responses, shifting the immune reaction toward an allergic phenotype. The abundant proteases and polysaccharides in these extracts are likely the primary sensitizing elements. They activate airway epithelial cells to release IL-33, IL-25, and TSLP, which then directly stimulate ILC2s in lung tissue. Activated ILC2s rapidly secrete large amounts of type 2 cytokines and subsequently promote Th2 cell activation, amplifying inflammation.

### 2.2 Key parameters and optimization of model construction

#### 2.2.1 Selection of experimental animals

BALB/c mice are recommended because they readily develop Th2-type immune responses, making them suitable for modeling human allergic asthma and ABPA. In contrast, C57BL/6 mice display a relative Th1 bias. Genetically modified strains can be used to define the functions of specific molecules or pathways (Wang et al., 2024). For example, hβcTg transgenic mice have been used to establish a model of *A. fumigatus*–induced chronic airway inflammation and fibrosis, as these mice respond to murine GM-CSF and IL-5 but not IL-3 (Mueller et al., 2009). Other studies have used CFTR^-^/^-^ mice and crude *A. fumigatus* protein extracts for sensitization and challenge, successfully reproducing ABPA-like immunopathology observed in patients with cystic fibrosis (Mueller et al., 2009).

#### 2.2.2 Exposure route and protocol

Filtrate extract–induced models commonly use intraperitoneal injection and intranasal instillation. Intraperitoneal injection induces systemic sensitization but does not necessarily produce prominent respiratory symptoms, so it is typically used during the sensitization phase. Intranasal instillation requires careful technique to avoid suffocation and may cause apoptosis of olfactory sensory neurons. In addition to these methods, some studies have applied oropharyngeal inoculation of *A. fumigatus* mycelial extract. Because conidia do not produce proteases and germinating spores have not yet developed hyphae, proteases are produced primarily by hyphae. Existing research indicates that intranasal instillation induces stronger pulmonary inflammatory infiltration and cytokine expression than aerosol inhalation. Mehlhop et al. established a chronic exposure model using intranasal instillation and demonstrated that significant airway eosinophilic inflammation and bronchial hyperreactivity can occur even in the absence of IgE (Mehlhop et al., 1997).

More recent approaches combine intraperitoneal sensitization with intranasal challenge, inducing both strong systemic immunity (high IgE levels) and representative local pulmonary pathology. However, these methods do not reflect natural airborne fungal exposure, because extracellular and intracellular antigens may be directly released into the suspension, altering both the concentration of soluble antigens and the physical or metabolic properties of the fungal particles.

#### 2.2.3 Selection of fungal strains

The choice of fungal strain is critical because it determines the potency of the filtrate extract. Extracts prepared from strains isolated from clinically sensitized patients or sensitized animals induce allergic responses more readily than extracts from laboratory strains.

Besides *A. fumigatus*, *A. alternata*—a potent environmental allergen—is also widely used to construct fungal sensitization models. In a murine model sensitized with *A. alternata* extract, Shi et al. used genetically engineered fungal strains to show that the pore-forming proteins Aeg-S and Aeg-L are key drivers of type 2 immunity (Shi et al., 2025). Geslewitz et al. used Alternaria filtrate and *A. fumigatus* extract to establish an acute Alternaria exposure model and a chronic *A. fumigatus* sensitization model, respectively (Geslewitz et al., 2018). The acute model used short-term, high-frequency exposure, whereas the chronic model used long-term, low-frequency exposure (up to 12 weeks) to mimic chronic or secondary immune activation. However, this chronic sensitization protocol included adjuvants, which may introduce non-specific inflammatory effects. Advances in genetic engineering have enabled more precise identification of strain-specific sensitizing factors. Namvar et al. constructed models using wild-type *A. fumigatus* culture filtrate (containing Asp f 5 and Asp f 13 proteases), a Δ5 mutant filtrate lacking Asp f 5, and a Δ13 mutant filtrate lacking Asp f 13 (Namvar et al., 2015). Their analysis showed that Asp f 5 and Asp f 13—particularly Asp f 13—are key mediators of airway inflammatory cell recruitment and remodeling but are not essential for AHR or Th2 cytokine production11. Notably, no adjuvants were used during sensitization in that study.

### 2.3 Disease phenotype induced by the model

Compared with spore models, filtrate extract–induced models exhibit stronger pathogenicity. Even without exogenous adjuvants, potent sensitization occurs regardless of the route of primary immunization. The toxins and enzymes present in the extract, including proteases, may function as natural adjuvants by damaging the airway epithelium and allowing antigens that are normally excluded to bypass the mucosal barrier (Kurup and Grunig, 2002; Grunig and Kurup, 2003).

These models consistently induce a Th2-type cytokine response characterized by high expression of IL-4, IL-5, and IL-13. They also lead to eosinophil accumulation, increased serum total IgE, elevated *A. fumigatus*-specific IgE/IgG levels, and AHR. Pathologically, filtrate extract–induced models reproduce key features of ABPA, including pulmonary eosinophilic inflammation, mucus hypersecretion, airway remodeling, and fibrosis (Namvar et al., 2015).

#### 2.3.1 Phenotypic comparison with the spore model

Comparisons between adult and neonatal mouse models show that both filtrate and spore exposure increase AHR, but the underlying immune responses differ. Filtrate models are dominated by eosinophilia and antibody responses, whereas spore models induce mixed neutrophilic–eosinophilic inflammation and can activate a Th17 response. Spore exposure significantly increases AHR in both age groups. Adult mice exhibit higher lymphocyte and IgG1 levels than neonatal mice, suggesting that immature neonatal immunity may increase susceptibility to allergic asthma. Eosinophil counts and IgE levels are similar across both models, indicating that despite strong Th2 induction, filtrate exposure does not elicit the Th17 and neutrophilic components triggered by spores. Findings from Castanhinha et al. and additional studies show that neonatal mouse models can effectively simulate pediatric fungal sensitization (Castanhinha et al., 2015).

#### 2.3.2 Application in modeling other fungal-associated airway diseases

Mulligan et al. established a mouse model of *A. fumigatus*-induced chronic rhinosinusitis (CRS) using BALB/c wild-type and C3aR^-^/^-^ mice (8 weeks old). Sensitization by intraperitoneal injection followed by intranasal challenge reproduced key features of human CRS with nasal polyps (CRSwNP), including local complement activation, type 2 inflammation, and polypoid lesions (Mulligan et al., 2018).

### 2.4 Advantages and limitations of the model

Currently, a central issue in AFAD research is distinguishing between transient fungal sensitization and disease persistence driven by sustained colonization. The filtrate extract model discussed in this section represents a typical model for the former. This model has several advantages. First, as noted above, it is simple and highly reproducible, making it one of the most widely used approaches, and it closely reproduces the core immunological features of human AFAD, including strong Th2 immune responses, eosinophil accumulation, elevated serum IgE, and airway hyperreactivity. Second, it allows study of early epithelial type 2 signaling without interference from live fungal infection, wherein proteases activate epithelial cells to release IL-33, IL-25, and TSLP, which then stimulate ILC2s (Kurup and Grunig, 2002; Grunig and Kurup, 2003). This pathway—from epithelial alarmins to ILC2 activation—is a key initiating event in fungal-induced allergic inflammation. Third, some studies have indicated that this model does not require exogenous adjuvants yet still produces potent sensitization, because fungal proteases can act as natural adjuvants. Therefore, to some extent it avoids confounding effects introduced by exogenous adjuvants (such as aluminum hydroxide), allowing a more direct assessment of fungal component-driven allergic responses.

Although filtrate extract models strongly induce sensitization, their heterogeneous composition makes it difficult to identify the active molecules responsible for immunogenicity. Preparation conditions such as temperature, extraction duration, and culture medium composition introduce variability, and repeated freeze–thaw cycles may inactivate proteases, cause denaturation, and increase the likelihood of non-specific inflammation. Intranasal instillation, intraperitoneal injection, and fluctuations in anesthesia depth can also lead to inconsistent dosing. This highlights the need for more precise delivery methods. Filtrate extract models do not fully replicate natural human exposure, which occurs through inhalation of dry fungal aerosols. Even so, these models allow non-invasive and repeatable procedures and avoid confounding effects from adjuvants. However, because of their strong sensitizing capacity, they may shift animals toward more invasive pulmonary pathology resembling pulmonary aspergillosis, likely reflecting the inherently high invasive potential of the extracts.

### 2.5 Perspective and future directions

A potential future strategy is to use filtrate extracts to disrupt the epithelial barrier and then induce sensitization with less invasive conidia. For example, sensitization with Cladosporium fulvum protease extract significantly enhances allergic airway inflammation in mice subsequently exposed to live spores.

## 3 Live spore inhalation models

### 3.1 Overview and fundamental principles

In addition to extract-based systems, live spore inhalation models are widely used. These models deliver viable fungal conidia to the respiratory tract by intranasal instillation or aerosol inhalation. They directly simulate human fungal sensitization–induced asthma. Their central principle is the repeated airway delivery of live, biologically active conidia. A major advantage of this approach is its close resemblance to natural inhalation exposure. It can reproduce transient airway colonization, sustained antigen release, and dynamic interactions between the host and fungi. This captures the full process of sensitization, chronic airway inflammation, and related physiological changes. Therefore, live spore inhalation models are regarded as a gold standard for studying fungal-associated allergic asthma, particularly chronic disease (Blease et al., 2002; Denis et al., 2007; Gao et al., 2009; Dorsam et al., 2010).

### 3.2 Key technical parameters and model construction

#### 3.2.1 Selection of fungal species and clinical relevance

The choice of fungal species is critical for constructing sensitization models. *A. fumigatus* is the most clinically relevant species because of its strong association with human allergic asthma, especially severe and fungal-sensitized forms (Blease et al., 2002; Gao et al., 2009). For example, the clinical isolate *A. fumigatus* W72310, obtained from the sputum of an ABPA patient, can persist in the lungs of immunocompetent mice as conidia for up to 21 days, providing sustained antigenic stimulation and mimicking chronic ABPA (Jones et al., 2021). Aspergillus niger has also been used to induce airway fungal infection and asthma-like pathology (Zeng et al., 2021). Cladosporium cladosporioides and *A. alternata (*Daines et al., 2021), recognized human respiratory allergens that do not cause pulmonary colonization or invasive infection, offer higher experimental safety and are suitable for allergic asthma models (Denis et al., 2007; Gao et al., 2009). Most studies employ laboratory strains such as *A. fumigatus* ATCC 13073 (Garth et al., 2018). These strains are cultured on solid media such as Potato Dextrose Agar or Sabouraud Dextrose Agar; conidia are collected by washing with PBS containing surfactant and filtered to remove hyphal fragments, producing a suspension of pure live conidia for animal use (Hogaboam et al., 2003).

#### 3.2.2 Core principle and necessity of using live spores

Spore viability is essential for initiating allergic responses. Live, dry conidia preserve the natural conformation and biological activity of surface pathogen-associated molecular patterns (PAMPs) (Lilly et al., 2012). Work by Nayak et al. showed that only live spores induce typical allergic inflammation. A key determinant is the exposure of β-glucan during germination, which is recognized by Dectin-1 and triggers a cascade of protease secretion and stage-specific allergen release. This process exacerbates inflammation through epithelial–immune cell interactions and metabolic or organelle stress (Lilly et al., 2012). *In vitro* studies by Lea Pylkkänen et al. demonstrated that live spores specifically activate innate immune cells such as macrophages, initiating pro-inflammatory cytokine and chemokine production (Pylkkänen et al., 2004). Rivera et al. further showed that live spores induce a strong Th1 response and IgG antibody production in murine lungs, whereas heat-killed spores elicit a Th2-biased response and fail to generate effective IgG (Rivera et al., 2005). Thus, live spores more accurately capture the immune spectrum that occurs during natural infection and sensitization and represent a physiologically relevant exposure material. During germination, new antigens (such as hyphal proteins) and PAMPs (such as β-glucan) become exposed, and proteases are released, all of which contribute to effective sensitization. These findings indicate that “spore activity” is an independent and critical factor in driving allergic sensitization (Houlder et al., 2025).

Importantly, fungal viability is not merely a prerequisite for sensitization but fundamentally dictates the polarization of the immune response. In stark contrast to the mixed immune phenotype seen with live spore models, transient exposure to inactivated spores or fungal extracts typically elicits a Th2-dominant classical allergic response with negligible IL-17 or IL-22 secretion (Rivera et al., 2005; Lilly et al., 2012), owing to the absence of dynamic PAMP exposure (such as β-glucan) and accompanying protease and metabolic activities during germination. Conversely, β-glucan exposed during live spore germination is recognized by Dectin-1, which specifically promotes Th17 differentiation and IL-17/IL-22 secretion, thereby shifting the immune phenotype from pure Th2 to a mixed Th2/Th17 pattern. Thus, fungal viability constitutes a core switch determining the immune endotype of these models. This addition mechanistically clarifies the concept of “fungal viability as a core switch of immune endotype.”

#### 3.2.3 Determination and optimization of key model parameters

For sensitization, “resting spores” are often used to simulate naturally inhaled allergens and to study the early mechanisms of sensitization. In contrast, “pre-swollen spores” are produced by incubating spores in culture medium at 37 C for several hours. This yields a pre-germination state characterized by water uptake, cell wall remodeling, and exposure of immunogenic components such as chitin. These spores generate stronger immune stimulation and are useful for studying immune regulation or inflammation exacerbation (Ellis et al., 2024).

The choice of animal strain should align with study objectives. BALB/c mice, which exhibit a Th2 bias, are well suited to allergic asthma models (Blease et al., 2002; Denis et al., 2007; Dorsam et al., 2010; Hoselton et al., 2010). C57BL/6 mice, which show a stronger Th17 tendency, are appropriate for investigating neutrophilic inflammation. However, strain selection is not absolute. Studies by Samarasinghe, Zeng, and colleagues show that typical Th2-type asthma phenotypes can be induced in C57BL/6 mice when sensitization and challenge protocols are sufficiently robust (Samarasinghe et al., 2010; Zeng et al., 2021). This illustrates that model design, including the choice of strain and challenge agent, strongly influences experimental outcomes. Transgenic strains such as humanized βc receptor mice or Stat6−/− mice enable analysis of specific molecular pathways and evaluation of therapeutic strategies (Blease et al., 2002; Wang et al., 2024). Age is also relevant: Daines et al. used 5-week-old and 7-day-old mice to model adult and neonatal life stages, respectively, to test age-dependent sensitization (Daines et al., 2021).

Exposure routes include intranasal instillation (Blease et al., 2002; Denis et al., 2007; Gao et al., 2009; Daines et al., 2021) and aerosol inhalation. Intranasal instillation is operationally simple. Aerosol inhalation exposes anesthetized mice to aerosolized live spores in a dedicated chamber, producing a more uniform pulmonary distribution and better approximating natural exposure. Cook et al. used intranasal instillation to establish a repetitive, low-dose *A. fumigatus* exposure model that generated mixed type 2 and type 17 inflammation, resembling severe asthma (Cook et al., 2025). Ellis and Steele et al. employed intratracheal delivery of a single high dose followed by repeated low-dose challenges to establish a chronic fungal asthma model (Lilly et al., 2012; Ellis et al., 2024). Inhalation dose standardization can be achieved by controlling airflow (2psi) and exposure duration (10 minutes) (Samarasinghe et al., 2010). Typical doses range from 10^4^ to 10^7^ spores per exposure (Gao et al., 2009). Exposure frequency and duration depend on research aims: acute models use short-term, high-frequency dosing (Zeng et al., 2021), whereas chronic models use long-term, low-frequency exposure (4–12 weeks) to study airway remodeling (Gao et al., 2009; Lilly et al., 2012).

Some protocols adopt a two-stage strategy of systemic sensitization followed by local challenge. Animals are first sensitized with antigen extract plus adjuvant (such as OVA or soluble *A. fumigatus* antigen) and then challenged with live spores to mirror progression from sensitization to disease (Blease et al., 2002; Gao et al., 2009; Dorsam et al., 2010; Hoselton et al., 2010). However, work by Denis and Zeng demonstrates that chronic exposure to natural mold spores alone, without any adjuvant or prior sensitization, can break tolerance and induce a complete asthma phenotype (Denis et al., 2007; Zeng et al., 2021). This non-pre-sensitized approach more closely simulates chronic fungal exposure in humans.

### 3.3 Disease phenotypic features induced by the model

#### 3.3.1 Airway inflammation and immune response

A defining feature of the live spore inhalation model is mixed granulocytic infiltration in the airways, with significant increases in eosinophils, lymphocytes, macrophages, and neutrophils in bronchoalveolar lavage fluid (Denis et al., 2007; Jones et al., 2021), with eosinophils comprising up to 75% of total BAL cells (Williams et al., 2026). Correspondingly, the adaptive immune system exhibits concurrent Th2 and Th17 activation: both Th2 and Th17 lymphocyte subsets accumulate in the lungs, and their cytokines, including IL-5, IL-13, and TGF-β, are elevated, whereas IFN-γ typically shows little change (Gao et al., 2009; Zeng et al., 2021). Notably, Jones et al. reported that during recall challenge the inflammatory response becomes further intensified and is accompanied by synchronous increases in Th1-type (IFN-γ) and Th2-type cytokines (Jones et al., 2021), highlighting the complexity of the induced immune network.

#### 3.3.2 Immunoglobulin response

In humoral immunity, model animals display hallmarks of fungal sensitization, including substantial increases in serum total IgE (Denis et al., 2007; Gao et al., 2009; Zeng et al., 2021) and elevated fungal-specific IgE and IgG1 levels (Denis et al., 2007). These responses provide essential immunological evidence consistent with allergic asthma.

#### 3.3.3 Lung function and airway pathological changes

Functionally and structurally, animals exhibit significant AHR together with typical airway remodeling. Remodeling features include goblet cell hyperplasia or metaplasia with increased mucus production, subepithelial collagen deposition, and smooth muscle thickening (Blease et al., 2002; Denis et al., 2007; Gao et al., 2009; Samarasinghe et al., 2010; Daines et al., 2021; Zeng et al., 2021). The severity of AHR often correlates with the extent of these structural abnormalities (Gao et al., 2009). Histological examination commonly shows germinating spores and hyphae in lung tissue, and chronic models may develop tertiary lymphoid structures and fibrotic lesions (Zeng et al., 2021). For example, the chronic fungal sensitization model established by Denis et al. produced sustained AHR even after exposure ceased, together with substantial collagen accumulation and elevated hydroxyproline, soluble collagen, and fibronectin. Histology confirmed obvious subepithelial fibrosis (Denis et al., 2007). In contrast, certain acute models (e.g., exposure to A. niger alone) can induce some remodeling features but do not generate fibrosis within the experimental timeframe (Zeng et al., 2021).

### 3.4 Key mechanistic insights

The live spore inhalation model is valued not only for accurately reproducing human fungal-sensitized asthma but also for revealing essential mechanisms underlying this complex immune disorder.

#### 3.4.1 Initiation of immune response: barrier disruption and innate recognition

Disease initiation begins with the interaction between live spores and the airway epithelial barrier. Fungal antigens have high protease activity, and live spores can secrete proteases, potentially through activation of protease-activated receptor 2 (PAR-2). These proteases disrupt epithelial integrity and stimulate the release of IL-33, TSLP, and IL-25, which initiates type 2 immunity (Denis et al., 2007). Recent studies have further elucidated the fine regulation of this process at the level of dendritic cell (DC) recognition, revealing that the initiation of allergic responses is highly dependent on fungal spore morphotype. Houlder et al. demonstrated that dendritic cells require at least 3 hours of Aspergillus fumigatus spore swelling to become effectively activated and initiate allergic airway inflammation, whereas resting or earlier-stage spores lack this capacity. Extending these findings, Cook et al. (2025) used single-cell approaches to show that in lung-draining lymph nodes, Mgl2^+^ conventional type 2 dendritic cells (cDC2s) are the key subset that selectively drives type 2, but not type 17, inflammation. At the same time, Dectin-1 recognition of β-glucan in the spore cell wall activates pathways that drive ILC3 and Th17 responses and promote neutrophilic inflammation (Denis et al., 2007; Zeng et al., 2021). Chatterjee et al. further demonstrated that the airway iron-rich microenvironment upregulates fungal protease expression via the transcription factor PrtT, thereby exacerbating epithelial barrier disruption and promoting Th2 inflammation (Chatterjee et al., 2026). These parallel innate mechanisms enable live spore inhalation to generate a stable mixed Th2/Th17 phenotype with neutrophilic inflammation and AHR, making it a suitable system for studying severe or fungal-sensitized asthma (Lilly et al., 2012).

#### 3.4.2 Chronicity and progression of the disease

Chronic disease progression depends on fungal persistence and the coordination of multiple signaling pathways. For instance, the prolonged persistence of the *A. fumigatus* W72310 strain in the lung provides sustained antigenic stimulation and supports chronic disease development (Jones et al., 2021). During chronic phases, regulatory networks become more complex. Steele et al. demonstrated that IL-22, induced through Dectin-1 signaling, is a key effector cytokine promoting excessive mucus secretion, chemokine production, and impaired lung function; neutralizing IL-22 directly improved lung function, indicating its therapeutic potential (Lilly et al., 2012). Compensatory mechanisms have also been identified. In Stat6 deficiency, IL-13 can still drive AHR and fibrosis via Stat6-independent pathways, challenging conventional assumptions about Th2-mediated disease (Blease et al., 2002).

#### 3.4.3 Revealing complexity: immune networks beyond convention

This model highlights synergistic interactions among multiple fungal components rather than a single dominant factor. Ellis et al. showed that chitin exposure alone does not induce AHR. Chitinase-mediated degradation of the spore cell wall may release additional highly immunogenic PAMPs. Dectin-1, for example, plays a central role in sensing β-glucan and driving inflammation and AHR (Ellis et al., 2024). Inhalation of live *A. fumigatus* spores has revealed the full pathogenic cascade, from Dectin-1 recognition of germinating spores to IL-33/ST2 signaling and metabolic dysregulation (Houlder et al., 2025), highlighting the complex immune circuitry of fungal asthma.

### 3.5 Model advantages and limitations

Within the spectrum of AFAD animal models, the model involving repeated inhalation of live spores currently best reflects the core clinical state of “fungal colonization-driven chronic disease.” This live spore inhalation model is highly clinically relevant and biologically authentic. By replicating natural aerosolized fungal exposure, the model reproduces complex human immune endotypes, including mixed Th2, Th17, and Th1 responses, as well as key pathological features. It is widely used to investigate host–fungus interactions and is particularly suited for studying chronicity, disease relapse, and steroid resistance (Denis et al., 2007; Samarasinghe et al., 2010).

Its major advantage is the ability to capture immune regulation driven by the biological activity of live fungi, which cannot be reproduced using dead spores (Pandey et al., 2013) or purified extracts (Samarasinghe et al., 2010). The model has clarified critical mechanisms, such as germination-driven Th17/neutrophilic inflammation (Daines et al., 2021). Compared with liquid-based instillation of spores, inhalation of dry spores directly from mature colonies better preserves surface hydrophobicity and antigenic integrity, more accurately reflecting human exposure (Daines et al., 2021). The approach also achieves good reproducibility using standardized spore culture systems and inhalation devices (Hoselton et al., 2010). Avoiding adjuvants eliminates non-specific immune activation and allows direct study of fungal pathogenicity (Denis et al., 2007; Daines et al., 2021).

However, the model has limitations. Technically, it requires BSL-2 or higher facilities, and standardizing spore preparation, nebulization efficiency, and precise inhaled doses remains challenging. Chronic models require long exposure periods (often more than 10 weeks), increasing cost and labor (Denis et al., 2007; Gao et al., 2009; Hoselton et al., 2010; Samarasinghe et al., 2010). Mechanistically, because live fungi activate many immune pathways simultaneously, assigning specific phenotypes to individual fungal components can be difficult (Ellis et al., 2024). In disease simulation, current models still cannot fully recapitulate all ABPA features, such as bronchocentric colonization (Hogaboam et al., 2003).

Future work should prioritize protocol standardization to improve dose precision; increased use of clinical isolates and genetically engineered fungi; integration with humanized mice or disease-relevant backgrounds to enhance translational value; and development of therapeutics targeting fungal virulence pathways (e.g., Dectin-1 signaling) (Denis et al., 2007; Gao et al., 2009; Samarasinghe et al., 2010). Expanding model applications, for example by using neonatal models to screen interventions that prevent early-life sensitization and airway remodeling (Daines et al., 2021), may further advance the field.

## 4 Combined allergen–fungal exposure models

### 4.1 Clinical background and modeling significance

Clinical evidence shows that patients with allergic airway diseases are rarely sensitized to a single allergen; multi-sensitization is far more common. Co-sensitization to HDM and environmental fungi (such as *A. alternata* and *A. fumigatus*) is strongly associated with greater asthma severity, poor symptom control, and increased risk of exacerbations.

This raises a key question: how do different allergens collectively contribute to severe disease phenotypes? Do the immune pathways they trigger act independently and additively, or do they interact synergistically?

To address this, researchers have developed combined allergen–fungal exposure models. These models reproduce the clinical “multiple hits” scenario, enabling detailed investigation of the “second hit” mechanisms underlying acute exacerbations and revealing how diverse allergens interact to produce complex immune responses. These findings offer important mechanistic insights with direct clinical relevance.

### 4.2 General principles of model design

Combined exposure models are generally built using two conceptual strategies. The first establishes a stable allergic airway environment using a primary allergen, such as house dust mite (HDM) or ovalbumin (OVA). Fungal components, such as fungal extracts or live spores, are then introduced to evaluate their effects under pre-existing sensitization. The second approach exposes animals to both the primary allergen and fungal components at the same time, allowing direct examination of concurrent airway challenges. Model parameters, including mouse strain, fungal form and dose, and exposure duration, require careful optimization to match the study objectives. Proper model design is essential for reproducibility and for accurate interpretation of results.

### 4.3 Model and mechanism analysis

#### 4.3.1 Acute exacerbation models

In one model, BALB/c mice were first sensitized intranasally with HDM extract for three weeks, establishing a Th2-dominant inflammatory background. The mice then received a single *A. alternata* extract challenge (Snelgrove et al., 2014). This protocol induced a rapid acute exacerbation phenotype characterized by steep increases in eosinophils and neutrophils in bronchoalveolar lavage fluid, elevated expression of mucus genes MUC5AC and MUC5B, and significant worsening of lung function. Serine protease activity in Alternaria was identified as a key trigger. It activated protease-activated receptor-2 and ATP signaling, rapidly inducing IL-33 release from airway epithelial cells and driving type 2 innate lymphoid cell activation together with a mixed inflammatory response (Snelgrove et al., 2014).

#### 4.3.2 Chronic exacerbation and immune tolerance breakdown

In an OVA-sensitized Wistar rat model, animals were exposed to aerosolized *A. fumigatus* spores for four weeks (Liu et al., 2014). Compared with rats exposed to OVA alone, those receiving combined exposure showed more pronounced AHR and eosinophilic inflammation. These effects were closely associated with increased prostaglandin D_2_ (PGD_2_) levels and upregulated expression of its receptor CRTH2 in lung tissue. Treatment with the CRTH2 antagonist OC000459 almost completely prevented inflammatory exacerbation, highlighting the central role of the PGD_2_/CRTH2 pathway in fungus-induced acute exacerbation (Liu et al., 2014).

Combined exposure models have also been used to investigate chronic asthma persistence and the breakdown of immune tolerance. In one study, BALB/c mice were co-sensitized intranasally with HDM, ragweed, and *A. fumigatus* extracts (without adjuvant), followed by long-term intranasal challenge (Goplen et al., 2009). This produced a chronic asthma phenotype that persisted even after allergen withdrawal, with sustained AHR, eosinophilic inflammation, mucus hypersecretion, and considerable airway remodeling, including smooth muscle thickening and collagen deposition. Mechanistically, this phenotype was driven by synergistic activation of dendritic cell p38-MAPK signaling by multiple allergens, leading to enhanced expression of costimulatory molecules, breakdown of immune tolerance, and persistent Th2 memory (Goplen et al., 2009). Notably, this model was insensitive to anti-IL-5 and anti-IL-13 therapy, thereby better reflecting characteristics of severe human asthma (Goplen et al., 2009).

#### 4.3.3 Airway remodeling

Long-term fungal exposure also significantly affects mucus secretion. In an OVA-sensitized Wistar rat model, 1–5 weeks of intranasal *A. fumigatus* live spore exposure significantly upregulated airway MUC5AC expression in a time-dependent manner (Gao et al., 2012). Increases in MUC5AC mRNA and protein levels occurred before overt goblet cell hyperplasia, and this upregulation was closely associated with elevated IL-13 in bronchoalveolar lavage fluid, indicating mediation by a Th2 pathway. The level of MUC5AC expression correlated positively with the severity of AHR, providing new evidence that fungal exposure exacerbates asthma by enhancing mucus hypersecretion (Gao et al., 2012).

#### 4.3.4 Treatment intervention

A therapeutic study compared dexamethasone and itraconazole in mice sensitized with HDM and challenged with *A. fumigatus* live spores (Matsuse et al., 2013). Dexamethasone significantly reduced IL-5, attenuated eosinophilic inflammation, and restored impaired alveolar macrophage phagocytic activity. Itraconazole reduced the neutrophil chemokine MIP-2 and dampened both eosinophilic and neutrophilic inflammation. However, neither treatment significantly improved mucus hypersecretion, highlighting the need for additional targeted therapies (Matsuse et al., 2013).

In another study, female BALB/c mice were sensitized intraperitoneally and challenged intranasally with HDM extract (Fukushima et al., 2010). Using the *A. fumigatus* MF-13 strain, mice were exposed intranasally to live or dead spores. In sensitized mice, live spores produced substantial neutrophil recruitment and elevated MIP-2, with spores detectable in lung tissue. Both live and dead spores enhanced Th2 inflammation and mucus secretion (Fukushima et al., 2010).

### 4.4 Experimental grouping and mechanism summary

#### 4.4.1 Technical points of model construction

Overall, combined allergen–fungal exposure models show strong clinical relevance and flexibility in experimental design. Most studies use 6–8-week-old BALB/c mice, although Wistar and SD rats are also used. Strain selection aligns with research objectives: BALB/c mice are suited to examining multi-sensitization and chronic inflammation, whereas rat models show stable AHR and are useful for studying acute exacerbation mechanisms. Basic asthma models fall into two categories: clinically relevant intranasal sensitization with HDM extract, which can be administered without adjuvant, and mechanistic OVA models using intraperitoneal sensitization followed by aerosol or intranasal challenge. Some studies employ multi-allergen exposure (HDM + ragweed + *A. fumigatus*), which can break immune tolerance without adjuvant when delivered intranasally over extended periods. Fungal exposure is selected according to study aims. Extracts of *A. alternata* and *A. fumigatus* (containing allergens such as Alt a 1) are used to dissect specific mechanisms, whereas live *A. fumigatus* spores better simulate clinical fungal colonization and acute exacerbations. Intranasal instillation is the primary exposure route, with some studies using aerosol inhalation for more physiological relevance. Criteria for successful asthma modeling include AHR, eosinophil or neutrophil infiltration in bronchoalveolar lavage fluid, characteristic pathological changes in lung tissue (e.g., HE staining), and increased expression of mucin genes MUC5AC and MUC5B. Successful fungal sensitization is confirmed by elevated serum IgE and fungus-specific IgE, and activation of fungal-related pathways such as PGD_2_/CRTH2 or p38-MAPK in lung tissue, ensuring that the model satisfies both asthma and fungal sensitization criteria.

#### 4.4.2 Typical experimental grouping scheme

In these studies, baseline controls typically receive PBS, and some designs include an unsensitized blank control to exclude background interference. Core experimental groups consist of single-exposure and combined-exposure groups. Single-exposure groups include animals exposed only to allergens (HDM, OVA, ragweed) or to fungal components (e.g., Alternaria/Aspergillus extract or live spores) and serve as comparators for combined-sensitization groups. Combined-exposure groups are the primary focus and include dual exposures (e.g., HDM + *A. fumigatus*), triple exposures (HDM + ragweed + *A. fumigatus*), and sequential or simultaneous protocols that mimic different clinical sensitization patterns. Intervention groups include targeted blockade or pharmacologic treatments directed at key pathways or cytokines (such as CRTH2, serine proteases, IL-33/IL-5/IL-13), including agents such as AEBSF, dexamethasone, and itraconazole. Drug-only controls are sometimes included. Collectively, the grouping strategies ensure rigorous comparisons and facilitate investigation of synergistic inflammatory mechanisms.

#### 4.4.3 Core mechanism of synergy

Fungi exacerbate allergic asthma through multiple mechanistic routes. *A. alternata* serine proteases activate PAR-2 and ATP signaling, stimulating IL-33 release and thereby activating ILC2s and driving acute Th2 inflammation and mucus secretion. *A. fumigatus* acts through several independent pathways: it enhances eosinophilic inflammation and Th2 cytokine production through the PGD_2_/CRTH2 axis; on an HDM-sensitized background, its spore components synergistically amplify Th2 responses, and live spores additionally induce MIP-2–associated neutrophilic inflammation; and chronic exposure elevates IL-13, driving MUC5AC expression and goblet cell hyperplasia. When *A. fumigatus* is combined with HDM and ragweed, dendritic cell p38-MAPK signaling is activated, with increased MHC II and CD40 expression, breaking immune tolerance and establishing persistent Th2 inflammation and airway remodeling.

### 4.5 Summary and prospect

Combined allergen–fungal exposure models, by simulating clinically common multi-sensitization, substantially advance understanding of the complexity of allergic airway diseases. These models form an important bridge between basic immunology and clinical practice. Future directions include developing more complex multi-exposure models (such as integrating HDM, fungi, and viral stimuli) to better approximate real-world exposure histories; using the models to test the efficacy of novel biologics or small-molecule therapies under complex inflammatory conditions; and integrating humanized mouse systems or organoid technologies to enable studies in experimental platforms with more human-like immune characteristics and tissue architecture. Such strategies will support the development of precision medicine approaches for severe allergic airway diseases.

## 5 Conclusion

**Figure 1:**
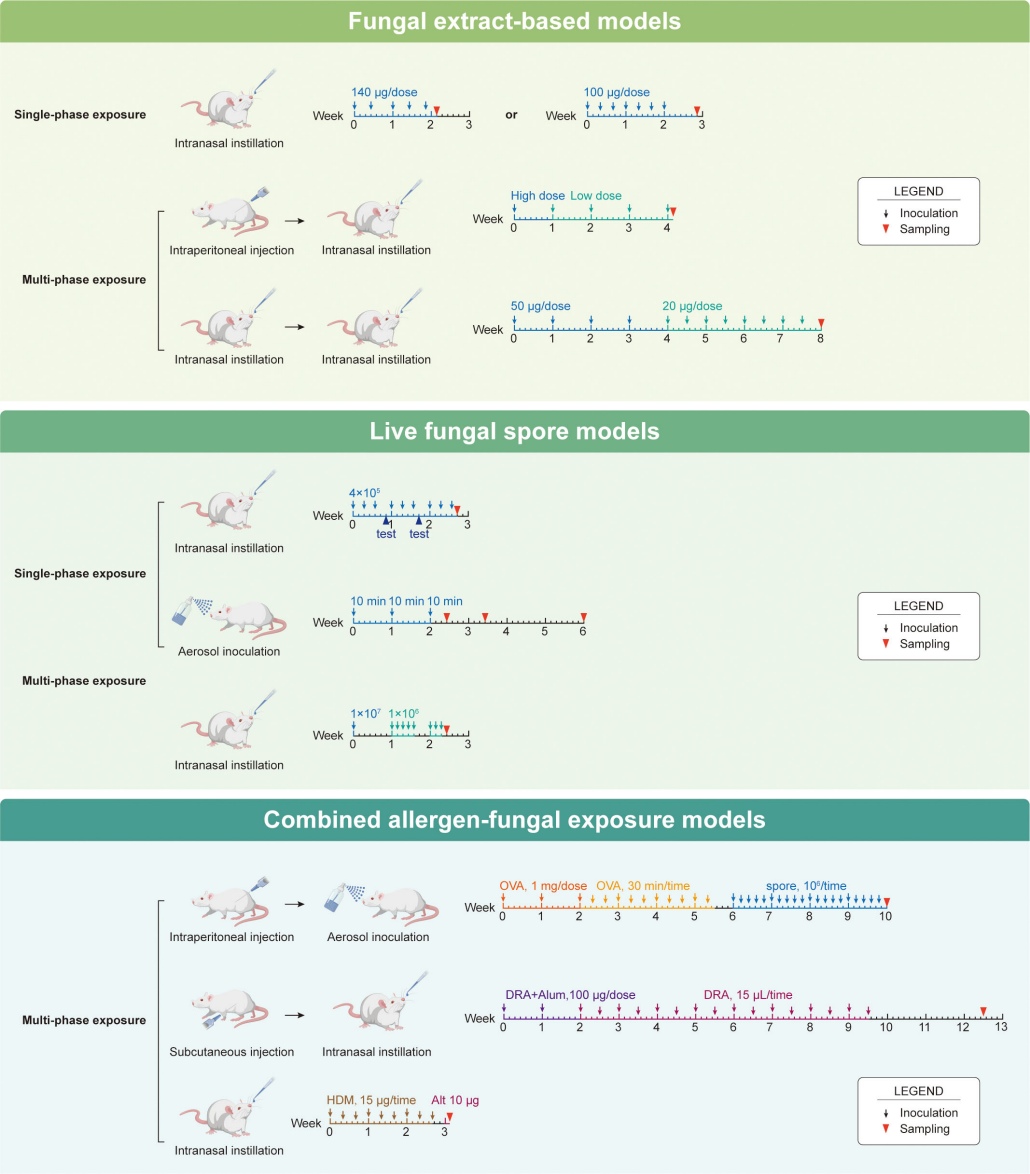
Classification and protocol comparison of mouse models of fungal sensitization.

**Figure 2:**
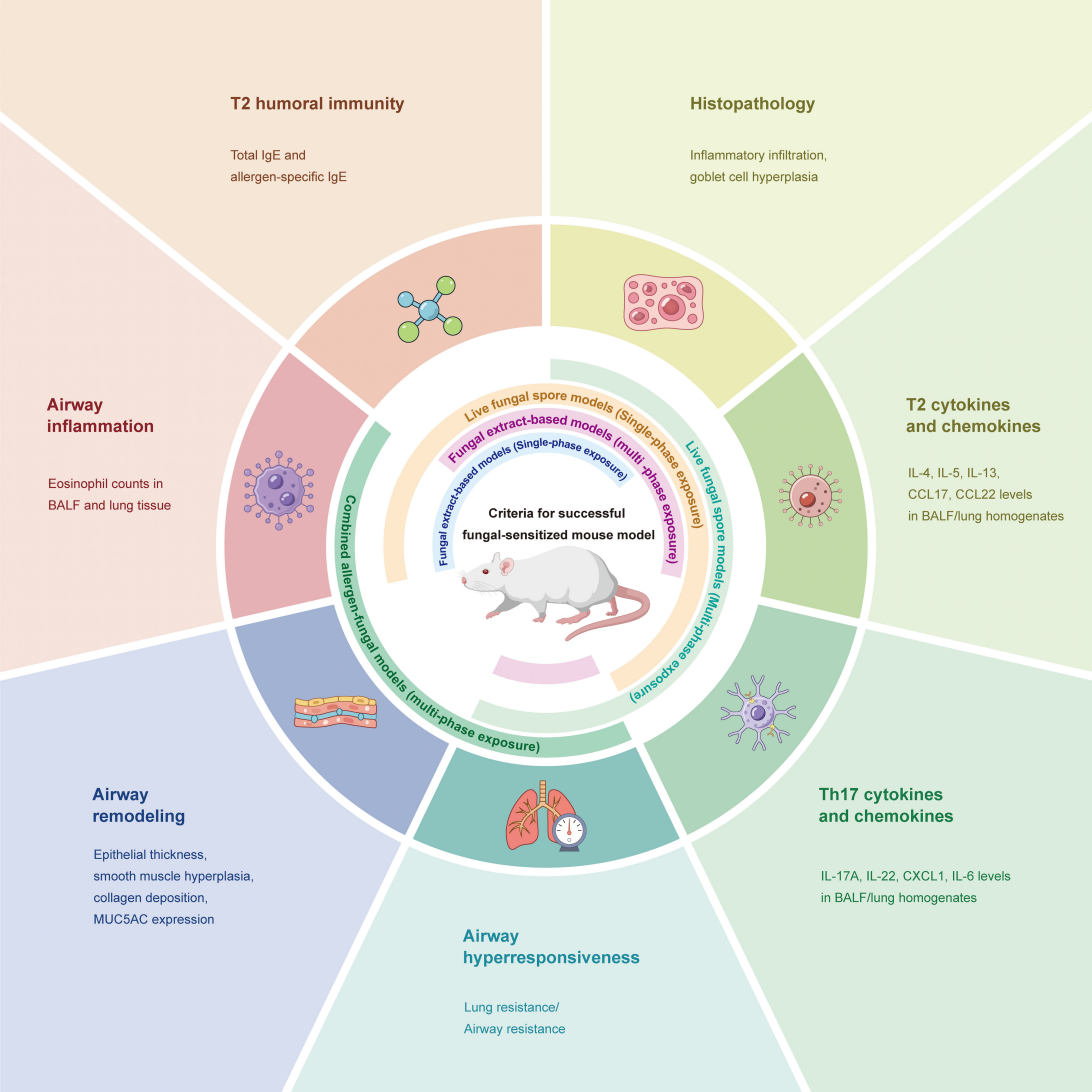
Criteria for a successful fungal-sensitized mouse model.

In conclusion, this review systematically describes three categories of fungal sensitization animal models—_those based on fungal extracts, live spores, and combined allergen exposure—and explicitly distinguishes them from the perspectives of “transient fungal sensitization” and “disease persistence driven by sustained colonization.” Although these models differ in sensitization protocols and exposure doses (**Figure 1**), collectively they cover the core pathological spectrum of human fungus-associated asthma, from acute sensitization to chronic disease persistence: transient sensitization models primarily reproduce airway hyperresponsiveness, eosinophilic inflammation, and type 2 immune initiation, whereas sustained colonization models further drive mixed Th17 inflammation and irreversible airway remodeling (**Figure 2**).

Fungal extract models, owing to their standardized composition and operational convenience, are particularly suitable for elucidating the sensitization mechanisms of individual fungal molecules. While they efficiently induce type 2 inflammation and airway hyperresponsiveness, the antigenic stimulation in the airways is transient and unsustainable because of the absence of live fungal components; thus, these models more closely approximate a transient fungal sensitization state. In contrast, live spore–based models simulate the dormancy–germination–hyphal transformation process of spores in the airways, leading to persistent epithelial barrier damage and continuous release of virulence factors such as fungal proteases. The ongoing presence of the pathogen sustains mixed type 2/Th17 inflammation, airway remodeling, and steroid hyporesponsiveness, thereby achieving disease chronicity driven by sustained airway colonization. This model more accurately reflects the dynamic host response following environmental exposure and therefore serves as an ideal platform for studying ABPA, chronic inflammation, and steroid-resistant phenotypes. Furthermore, the combinatorial allergen exposure model leverages the synergistic effects of multiple allergens to overcome immune tolerance induced by single antigens, thereby better mimicking the clinically prevalent state of polysensitization and the features of difficult-to-treat asthma (**Table 2**).

**Table 2:** Multidimensional comparison of immunopathological features across different animal models of fungal sensitization.

Fungal viability	Fungal extract model		Live spore model		Combined allergen-fungal exposure model	
	No		Yes		No (if extract-based)/Yes (if live spore-based)	
Exposure chronicity	Short-term	Long-term	Short-term	Long-term	Short-term	Long-term
Immune Endotype	T2-dominant	T2-dominant with Th17 involvement	Th1-dominant	Mixed T2/Th17	T2-dominant	T2-dominant with Th17 involvement
Cellular Inflammation Type	Eosinophil-predominant inflammation	Mixed granulocytic infiltration: predominantly eosinophils and neutrophils, with macrophages, lymphocytes, and mast cells	Mixed neutrophils, eosinophils, and inflammatory monocytes	Mixed granulocytic infiltration (eosinophils more prominent, with neutrophils and lymphocytes)	Eosinophil-predominant	Mixed granulocytic infiltration (eosinophils more prominent, with neutrophils and lymphocytes)
T2 Features	+++	+++	±	++~++++	++++	++++
Th17 Features	–	-~++	NA	++~+++	NA	+~++
Airway Remodeling Severity	NA	++~++++	±	+++~++++	±	+++~++++
Corresponding Clinical Stage	Acute invasive infection phase	Chronic allergic fungal inflammation with remodeling	Initial innate immune sensing and early sensitization transition	Chronic fungal allergic asthma (e.g., ABPA)	Fungal-induced acute asthma exacerbation (non-sensitized, non-chronic colonization phase)	Chronic severe asthma

NA, Not reported in the literature; “–” = negative or none; “±” = weakly positive or uncertain; “+” = positive, with the number of “+” signs (from + to ++++) indicating the severity of the feature (more signs = greater severity).

Research based on these models has not only established a reliable preclinical platform for evaluating novel biologics, such as anti-IL-33 monoclonal antibodies and CRTH2 antagonists, but has also laid a theoretical foundation for identifying populations at high risk of fungal sensitization and for developing therapeutic strategies targeting epithelial injury pathways. Future efforts should focus on establishing standardized modeling protocols and integrating organoid technology and humanized mouse models to further enhance the clinical translational value across the full continuum from “sensitization” to “disease persistence”.
